# 3-(4-Fluoro­phenyl­sulfin­yl)-5-iodo-2-methyl-1-benzofuran

**DOI:** 10.1107/S1600536810024931

**Published:** 2010-06-30

**Authors:** Hong Dae Choi, Pil Ja Seo, Byeng Wha Son, Uk Lee

**Affiliations:** a Department of Chemistry, Dongeui University, San 24 Kaya-dong Busanjin-gu, Busan 614-714, Republic of Korea; b Department of Chemistry, Pukyong National University, 599-1 Daeyeon 3-dong, Nam-gu, Busan 608-737, Republic of Korea

## Abstract

In the title compound, C_15_H_10_FIO_2_S, the O atom and the 4-fluoro­phenyl group of the 4-fluoro­phenyl­sulfinyl substituent are located on opposite sides of the plane through the benzofuran fragment; the 4-fluoro­phenyl ring is nearly perpendicular to this plane, making a dihedral angle of 83.37 (7)°. The crystal structure is stabilized by weak inter­molecular C—H⋯O hydrogen bonds and an I⋯O inter­action [I⋯O = 3.255 (2) Å]. The crystal structure also exhibits inter­molecular C—F⋯π inter­actions [3.068 (2) Å], and aromatic π–π inter­actions between the furan and benzene rings of neighbouring benzofuran fragments [centroid–centroid distance = 3.636 (2) Å].

## Related literature

For the crystal structures of similar 3-(4-fluoro­phenyl­sulfin­yl)-2-methyl-1-benzofuran derivatives, see: Choi *et al.* (2010**a* ▶,*b* ▶,c* ▶). For the pharmacological activity of benzofuran compounds, see: Aslam *et al.* (2006 ▶); Galal *et al.* (2009 ▶); Khan *et al.* (2005 ▶). For natural products with benzofuran rings, see: Akgul & Anil (2003 ▶); Soekamto *et al.* (2003 ▶). For a review of halogen bonding, see: Politzer *et al.* (2007 ▶).
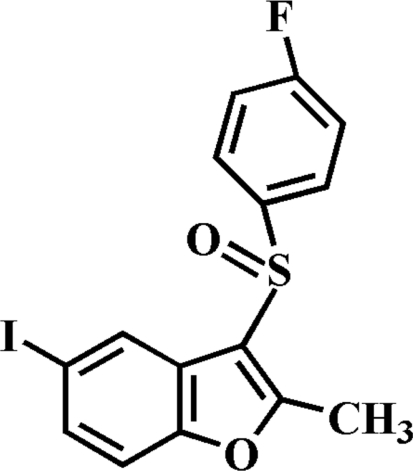


## Experimental

### 

#### Crystal data

C_15_H_10_FIO_2_S
*M* *_r_* = 400.19
Monoclinic, 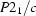

*a* = 13.1665 (4) Å
*b* = 11.4338 (4) Å
*c* = 9.9296 (3) Å
β = 107.181 (1)°
*V* = 1428.13 (8) Å^3^
*Z* = 4
Mo *K*α radiation
μ = 2.40 mm^−1^
*T* = 173 K
0.27 × 0.24 × 0.20 mm

#### Data collection

Bruker SMART APEXII CCD diffractometer
Absorption correction: multi-scan (*SADABS*; Bruker, 2009 ▶) *T*_min_ = 0.565, *T*_max_ = 0.648
12825 measured reflections
3297 independent reflections
2990 reflections with *I* > 2σ(*I*)
*R*_int_ = 0.031

#### Refinement

*R*[*F* ^2^ > 2σ(*F* ^2^)] = 0.029
*wR*(*F* ^2^) = 0.077
*S* = 1.11
3297 reflections
182 parameters
H-atom parameters constrained
Δρ_max_ = 0.58 e Å^−3^
Δρ_min_ = −1.56 e Å^−3^

### 

Data collection: *APEX2* (Bruker, 2009 ▶); cell refinement: *SAINT* (Bruker, 2009 ▶); data reduction: *SAINT*; program(s) used to solve structure: *SHELXS97* (Sheldrick, 2008 ▶); program(s) used to refine structure: *SHELXL97* (Sheldrick, 2008 ▶); molecular graphics: *ORTEP-3* (Farrugia, 1997 ▶) and *DIAMOND* (Brandenburg, 1998 ▶); software used to prepare material for publication: *SHELXL97*.

## Supplementary Material

Crystal structure: contains datablocks global, I. DOI: 10.1107/S1600536810024931/cs2129sup1.cif

Structure factors: contains datablocks I. DOI: 10.1107/S1600536810024931/cs2129Isup2.hkl

Additional supplementary materials:  crystallographic information; 3D view; checkCIF report

## Figures and Tables

**Table 1:** Hydrogen-bond geometry (Å, °)

*D*—H⋯*A*	*D*—H	H⋯*A*	*D*⋯*A*	*D*—H⋯*A*
C5—H5⋯O1^i^	0.93	2.55	3.472 (3)	170
C9—H9*C*⋯O2^ii^	0.96	2.53	3.277 (3)	135

Symmetry codes: (i) 
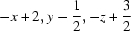
; (ii) 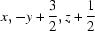.

**Fig. 1.**
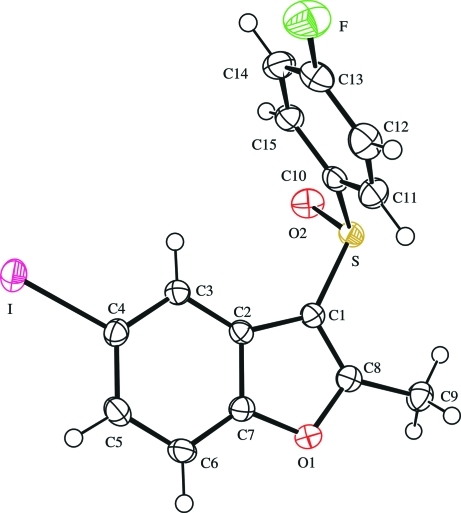
The molecular structure of the title compound with the atom numbering scheme. Displacement ellipsoids are drawn at the 50% probability level. H atoms are presented as a small spheres of arbitrary radius.

**Fig. 2.**
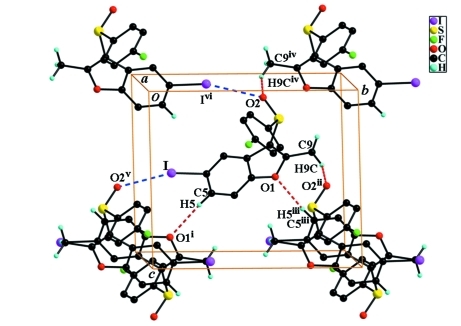
C—H···O and I···O interactions (dotted lines) in the crystal structure of the title compound. [Symmetry codes: (i) - x + 2, y - 1/2, - z + 3/2; (ii) x, - y + 3/2, z + 1/2; (iii) - x + 2, y + 1/2, - z + 3/2; (iv) x, - y + 3/2, z - 1/2; (v) x, - y + 1/2, z + 1/2; (vi) x, - y + 1/2, z - 1/2.]

**Fig. 3.**
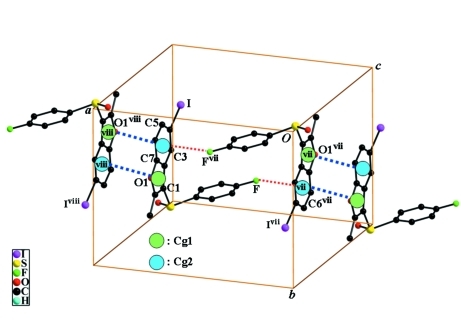
C—F···π and π–π interactions (dotted lines) in the crystal structure of the title compound. Cg denotes the ring centroid. [Symmetry codes: (vii) - x + 1, - y +1 , - z + 1; (viii) - x + 2, - y + 1, - z + 1.]

## supplementary crystallographic information

### Comment

Molecules containing benzofuran skeleton show variouspharmacological properties such as antifungal (Aslam *et al.*,  2006), antitumor and antiviral (Galal *et al.*., 2009), antimicrobial (Khan *et al.*., 2005) activity, and these compounds widely occur in nature (Akgul & Anil, 2003; Soekamto *et al.*., 2003). As a part of our ongoing studies of the effect of side chain substituents on the solid state structures of 3-(4-fluorophenylsulfinyl)-2-methyl-1-benzofuran analogues (Choi *et al.*., 2010*a,b,c*), we report the crystal structure of the title compound (Fig. 1).

The benzofuran unit is essentially planar, with a mean deviation of 0.008 (2) Å from the least-squares plane defined by the nine constituent atoms. The 4-fluorophenyl ring is almost perpendicular to the plane of the benzofuran fragment [83.37 (7)°] and is tilted slightly towards it. The crystal packing (Fig. 2) is stabilized by weak intermolecular C—H···O hydrogen bonds; the first one between the benzene H atom and the furan O atom with a C5—H5···O1^i^, and the second one between the methyl H atom and the oxygen of the S═O unit, with a C9—H9C···O2^ii^, respectively (Table 1). The molecular packing (Fig. 2) is also stabilized by an I···O halogen bonding between the iodine and the oxygen of the S═O unit [I···O2^v^ = 3.255 (2) Å; C4—I···O2^v^ = 164.42 (8)°] (Politzer *et al.*,  2007). The crystal packing (Fig. 3) also exhibits intermolecular C—F···π interactions between the fluorine and the benzene ring of an adjacent benzofuran system, with a C13—F···Cg2^vii^ distance of 3.068 (2) Å (Cg2 is the centroid of the C2–C7 benzene ring), and aromatic π–π interactions between the furan and the benzene rings of the adjacent benzofuran systems, with a Cg1···Cg2^viii^ distance of 3.636 (2) Å (Cg1 is the centroid of the C1/C2/C7/O1/C8 furan ring).

### Experimental

77% 3-Chloroperoxybenzoic acid (166 mg, 1.0 mmol) was added in small portions to a stirred solution of 3-(4-fluorophenylsulfanyl)–5-iodo-2-methyl-1-benzofuran (346 mg, 0.9 mmol) in dichloromethane (30 mL) at 273 K. After being stirred at room temperature for 3h, the mixture was washed with saturated sodium bicarbonate solution and the organic layer was separated, dried over magnesium sulfate, filtered and concentrated at reduced pressure. The residue was purified by column chromatography (hexane–ethyl acetate, 1:1 v/v) to afford the title compound as a colorless solid [yield 77%, m.p. 428–429 K; *R*_f_ = 0.64 (hexane–ethyl acetate, 1:1 v/v)]. Single crystals suitable for X-ray diffraction were prepared by slow evaporation of a solution of the title compound in benzene at room temperature.

### Refinement

All H atoms were positioned geometrically and refined using a riding model, with C—H = 0.93 Å for aryl and 0.96 Å for methyl H atoms. *U*_iso_(H) = 1.2*U*_eq_(C)  for aryl and 1.5*U*_eq_(C) for methyl H atoms.

### Crystal data

**Table:**

C_15_H_10_FIO_2_S	*F*(000) = 776
*M**_r_* = 400.19	*D*_x_ = 1.861 Mg m^−^^3^
Monoclinic, *P*2_1_/*c*	Mo *K*α radiation, λ = 0.71073 Å
Hall symbol: -P 2ybc	Cell parameters from 8763 reflections
*a* = 13.1665 (4) Å	θ = 2.4–27.6°
*b* = 11.4338 (4) Å	µ = 2.40 mm^−^^1^
*c* = 9.9296 (3) Å	*T* = 173 K
β = 107.181 (1)°	Block, colourless
*V* = 1428.13 (8) Å^3^	0.27 × 0.24 × 0.20 mm
*Z* = 4	

### Data collection

**Table:**

Bruker SMART APEXII CCD diffractometer	3297 independent reflections
Radiation source: rotating anode	2990 reflections with *I* > 2σ(*I*)
graphite multilayer	*R*_int_ = 0.031
Detector resolution: 10.0 pixels mm^-1^	θ_max_ = 27.6°, θ_min_ = 1.6°
φ and ω scans	*h* = −15→17
Absorption correction: multi-scan (*SADABS*; Bruker, 2009)	*k* = −14→14
*T*_min_ = 0.565, *T*_max_ = 0.648	*l* = −12→12
12825 measured reflections	

### Refinement

**Table:**

Refinement on *F*^2^	Primary atom site location: structure-invariant direct methods
Least-squares matrix: full	Secondary atom site location: difference Fourier map
*R*[*F*^2^ > 2σ(*F*^2^)] = 0.029	Hydrogen site location: difference Fourier map
*wR*(*F*^2^) = 0.077	H-atom parameters constrained
*S* = 1.11	*w* = 1/[σ^2^(*F*_o_^2^) + (0.0381*P*)^2^ + 1.2193*P*] where *P* = (*F*_o_^2^ + 2*F*_c_^2^)/3
3297 reflections	(Δ/σ)_max_ < 0.001
182 parameters	Δρ_max_ = 0.57 e Å^−^^3^
0 restraints	Δρ_min_ = −1.56 e Å^−^^3^

### Special details

**Table:**

Geometry. All esds (except the esd in the dihedral angle between two l.s. planes) are estimated using the full covariance matrix. The cell esds are taken into account individually in the estimation of esds in distances, angles and torsion angles; correlations between esds in cell parameters are only used when they are defined by crystal symmetry. An approximate (isotropic) treatment of cell esds is used for estimating esds involving l.s. planes.
Refinement. Refinement of F^2^ against ALL reflections. The weighted R-factor wR and goodness of fit S are based on F^2^, conventional R-factors R are based on F, with F set to zero for negative F^2^. The threshold expression of F^2^ > 2sigma(F^2^) is used only for calculating R-factors(gt) etc. and is not relevant to the choice of reflections for refinement. R-factors based on F^2^ are statistically about twice as large as those based on F, and R- factors based on ALL data will be even larger.

### Fractional atomic coordinates and isotropic or equivalent isotropic displacement parameters (Å^2^)

**Table:**

	*x*	*y*	*z*	*U*_iso_*/*U*_eq_	
I	0.774810 (15)	0.162106 (16)	0.53507 (2)	0.03259 (8)	
S	0.70499 (5)	0.67499 (6)	0.22747 (6)	0.02368 (14)	
F	0.31812 (15)	0.49689 (19)	0.3339 (2)	0.0480 (5)	
O1	0.94047 (15)	0.67042 (15)	0.57609 (19)	0.0249 (4)	
O2	0.71831 (16)	0.60325 (19)	0.10757 (19)	0.0318 (4)	
C1	0.8028 (2)	0.6377 (2)	0.3837 (2)	0.0213 (5)	
C2	0.82677 (18)	0.5281 (2)	0.4598 (2)	0.0195 (5)	
C3	0.78672 (19)	0.4142 (2)	0.4425 (2)	0.0219 (5)	
H3	0.7309	0.3931	0.3643	0.026*	
C4	0.8338 (2)	0.3340 (2)	0.5469 (3)	0.0233 (5)	
C5	0.9195 (2)	0.3625 (2)	0.6643 (3)	0.0263 (5)	
H5	0.9488	0.3058	0.7318	0.032*	
C6	0.9608 (2)	0.4746 (2)	0.6804 (3)	0.0259 (5)	
H6	1.0184	0.4951	0.7567	0.031*	
C7	0.91229 (19)	0.5544 (2)	0.5777 (2)	0.0214 (5)	
C8	0.8732 (2)	0.7187 (2)	0.4571 (3)	0.0229 (5)	
C9	0.8903 (2)	0.8441 (2)	0.4345 (3)	0.0292 (6)	
H9A	0.8451	0.8675	0.3436	0.044*	
H9B	0.9632	0.8567	0.4387	0.044*	
H9C	0.8735	0.8896	0.5064	0.044*	
C10	0.59180 (19)	0.6152 (2)	0.2691 (2)	0.0226 (5)	
C11	0.5564 (2)	0.6657 (2)	0.3746 (3)	0.0298 (6)	
H11	0.5945	0.7263	0.4293	0.036*	
C12	0.4639 (2)	0.6250 (3)	0.3973 (3)	0.0330 (6)	
H12	0.4393	0.6570	0.4679	0.040*	
C13	0.4092 (2)	0.5362 (3)	0.3133 (3)	0.0310 (6)	
C14	0.4427 (2)	0.4850 (3)	0.2094 (3)	0.0315 (6)	
H14	0.4039	0.4247	0.1549	0.038*	
C15	0.5360 (2)	0.5251 (2)	0.1870 (3)	0.0267 (5)	
H15	0.5607	0.4916	0.1173	0.032*	

### Atomic displacement parameters (Å^2^)

**Table:**

	*U*^11^	*U*^22^	*U*^33^	*U*^12^	*U*^13^	*U*^23^
I	0.03251 (12)	0.02096 (12)	0.04268 (13)	0.00056 (6)	0.00859 (9)	0.00097 (7)
S	0.0267 (3)	0.0237 (3)	0.0189 (3)	0.0016 (2)	0.0038 (2)	0.0032 (2)
F	0.0320 (9)	0.0613 (13)	0.0562 (11)	−0.0066 (9)	0.0216 (8)	−0.0055 (10)
O1	0.0236 (9)	0.0244 (10)	0.0240 (8)	−0.0017 (7)	0.0031 (7)	−0.0028 (7)
O2	0.0364 (10)	0.0411 (12)	0.0203 (8)	−0.0014 (9)	0.0122 (8)	−0.0030 (8)
C1	0.0214 (11)	0.0211 (12)	0.0208 (11)	0.0004 (9)	0.0056 (9)	0.0009 (9)
C2	0.0191 (11)	0.0211 (12)	0.0182 (10)	0.0021 (9)	0.0055 (8)	0.0003 (9)
C3	0.0219 (11)	0.0216 (12)	0.0208 (11)	0.0012 (9)	0.0043 (9)	−0.0012 (9)
C4	0.0250 (12)	0.0193 (13)	0.0258 (12)	0.0005 (9)	0.0079 (10)	−0.0022 (9)
C5	0.0293 (13)	0.0265 (13)	0.0215 (11)	0.0076 (11)	0.0050 (10)	0.0024 (10)
C6	0.0243 (12)	0.0286 (14)	0.0206 (11)	0.0034 (10)	0.0004 (9)	−0.0028 (10)
C7	0.0206 (11)	0.0230 (12)	0.0207 (10)	0.0009 (9)	0.0064 (9)	−0.0027 (9)
C8	0.0231 (12)	0.0247 (13)	0.0220 (11)	0.0013 (10)	0.0085 (9)	−0.0013 (9)
C9	0.0294 (14)	0.0238 (14)	0.0353 (14)	−0.0034 (10)	0.0111 (11)	−0.0010 (10)
C10	0.0234 (12)	0.0233 (13)	0.0183 (10)	0.0050 (10)	0.0017 (9)	0.0027 (9)
C11	0.0339 (15)	0.0285 (15)	0.0256 (12)	0.0020 (11)	0.0067 (11)	−0.0051 (10)
C12	0.0354 (15)	0.0368 (16)	0.0295 (13)	0.0068 (13)	0.0138 (11)	−0.0024 (12)
C13	0.0221 (12)	0.0380 (16)	0.0324 (13)	0.0038 (11)	0.0074 (10)	0.0035 (12)
C14	0.0272 (13)	0.0327 (15)	0.0319 (13)	−0.0027 (11)	0.0045 (11)	−0.0071 (11)
C15	0.0260 (12)	0.0295 (14)	0.0225 (11)	0.0034 (10)	0.0039 (10)	−0.0049 (10)

### Geometric parameters (Å, °)

**Table:**

I—C4	2.104 (2)	C6—C7	1.376 (4)
I—O2^i^	3.255 (2)	C6—H6	0.9300
S—O2	1.4981 (19)	C8—C9	1.478 (4)
S—C1	1.751 (2)	C9—H9A	0.9600
S—C10	1.795 (3)	C9—H9B	0.9600
F—C13	1.351 (3)	C9—H9C	0.9600
O1—C8	1.366 (3)	C10—C15	1.383 (4)
O1—C7	1.379 (3)	C10—C11	1.391 (4)
C1—C8	1.360 (4)	C11—C12	1.383 (4)
C1—C2	1.449 (3)	C11—H11	0.9300
C2—C3	1.396 (3)	C12—C13	1.375 (4)
C2—C7	1.396 (3)	C12—H12	0.9300
C3—C4	1.386 (3)	C13—C14	1.368 (4)
C3—H3	0.9300	C14—C15	1.389 (4)
C4—C5	1.401 (4)	C14—H14	0.9300
C5—C6	1.383 (4)	C15—H15	0.9300
C5—H5	0.9300		
			
C4—I—O2^i^	164.42 (8)	C1—C8—C9	133.5 (2)
O2—S—C1	110.03 (12)	O1—C8—C9	115.8 (2)
O2—S—C10	105.95 (12)	C8—C9—H9A	109.5
C1—S—C10	98.44 (11)	C8—C9—H9B	109.5
C8—O1—C7	106.94 (19)	H9A—C9—H9B	109.5
C8—C1—C2	107.5 (2)	C8—C9—H9C	109.5
C8—C1—S	121.0 (2)	H9A—C9—H9C	109.5
C2—C1—S	131.48 (19)	H9B—C9—H9C	109.5
C3—C2—C7	119.1 (2)	C15—C10—C11	121.0 (3)
C3—C2—C1	136.5 (2)	C15—C10—S	118.73 (19)
C7—C2—C1	104.4 (2)	C11—C10—S	120.1 (2)
C4—C3—C2	117.1 (2)	C12—C11—C10	119.3 (3)
C4—C3—H3	121.4	C12—C11—H11	120.3
C2—C3—H3	121.4	C10—C11—H11	120.3
C3—C4—C5	122.8 (2)	C13—C12—C11	118.6 (3)
C3—C4—I	120.02 (19)	C13—C12—H12	120.7
C5—C4—I	117.21 (19)	C11—C12—H12	120.7
C6—C5—C4	120.3 (2)	F—C13—C14	118.1 (3)
C6—C5—H5	119.9	F—C13—C12	118.9 (3)
C4—C5—H5	119.9	C14—C13—C12	123.0 (3)
C7—C6—C5	116.6 (2)	C13—C14—C15	118.5 (3)
C7—C6—H6	121.7	C13—C14—H14	120.8
C5—C6—H6	121.7	C15—C14—H14	120.8
C6—C7—O1	125.4 (2)	C10—C15—C14	119.5 (2)
C6—C7—C2	124.1 (2)	C10—C15—H15	120.2
O1—C7—C2	110.5 (2)	C14—C15—H15	120.2
C1—C8—O1	110.7 (2)		

Symmetry codes: (i) *x*, −*y*+1/2, *z*+1/2.

### Hydrogen-bond geometry (Å, °)

**Table:**

*D*—H···*A*	*D*—H	H···*A*	*D*···*A*	*D*—H···*A*
C5—H5···O1^ii^	0.93	2.55	3.472 (3)	170
C9—H9C···O2^iii^	0.96	2.53	3.277 (3)	135

Symmetry codes: (ii) −*x*+2, *y*−1/2, −*z*+3/2; (iii) *x*, −*y*+3/2, *z*+1/2.
